# Selection of appropriate reference genes in Apoe^-/-^ mouse brain

**DOI:** 10.1016/j.ibneur.2026.04.003

**Published:** 2026-04-24

**Authors:** Naz Mengi Camur, Busranur Seker, Fulya Kizildag, Tulin Yanik, Michelle M. Adams

**Affiliations:** a Department of Biological Sciences, Middle East Technical University, Cankaya, Ankara 06800, Türkiye; b Bilkent University, National Nanotechnology Research Center (UNAM), Ankara, Türkiye; c Bilkent University, Department of Neuroscience, Ankara, Türkiye; d Bilkent University, Department of Psychology, Ankara, Türkiye

**Keywords:** RT-qPCR, Reference Genes, Cerebral Cortex, Hippocampus, Hypothalamus, Apoe-/- Mice, Western Diet

## Abstract

**Background:**

Reverse transcription quantitative real-time polymerase chain reaction is the gold standard for gene expression quantification. Yet, this method’s accuracy heavily depends on choosing appropriate reference genes for data normalization. Reference genes must display stable expression levels across biological and experimental conditions to ensure accurate and meaningful results.

**New method:**

To address this problem, expression stability of six frequently used reference genes: *Actb*, *Gapdh*, *Rpl13a*, *Rplp0,* Hprt1*,* and *Ywhaz* in the cerebral cortex, hippocampus, and hypothalamus of C57BL/6 and Apoe^⁻/⁻^ mice given a chow or Western diet was evaluated using RefFinder, which utilizes four commonly used algorithms: the comparative ∆Ct method, BestKeeper, NormFinder, and geNorm. Additionally, the geometric mean of the two most stable genes was used to normalize the expression of the others to test the variability of less stable genes across brain regions, genotypes, and dietary conditions.

**Results:**

Results demonstrated that reference genes were the least stable in the hypothalamus, and the comprehensive ranking of the reference genes differed between the cerebral cortex and the hypothalamus. Notably, *Hprt1* in the cerebral cortex and *Actb* in the hypothalamus showed significant changes by diet and genotype.

**Comparison with existing methods:**

Reference gene stability is often assessed using individual algorithms like ∆Ct, BestKeeper, NormFinder, or geNorm. These algorithms utilize mathematical models and assumptions***.*** The combined RefFinder ranking provided a more robust evaluation, emphasizing subtle differences in gene stability across experimental conditions for more accurate reference gene selection***.***

**Conclusion:**

These results underline the importance of validating gene stability under specific experimental conditions.

## Highlights

• Reference gene stability varies across the cerebral cortex, hippocampus, and hypothalamus
• *Gapdh* and *Rpl13a* are the most stable genes in the cerebral cortex and hypothalamus
• *Hprt1* and *Actb* are the least stable genes in multiple brain regions
• Diet and genotype affect the stability of reference genes differently across brain regions

## 1 Introduction

Reverse transcription quantitative real-time polymerase chain reaction (RT-qPCR) is a widespread technique described as the gold standard for analyzing total RNA expression levels (VanGuilder et al., 2008). However, the accuracy of this technique relies on selecting the appropriate reference genes, which will be used in the normalization of target genes to measure their relative expression (Kozera and Rapacz, 2013). The expression levels of reference genes must remain unchanged through different biological and experimental conditions to obtain accurate results. Therefore, the precision of the normalization method is very critical, since the calculations of the relative expression of target genes will be directly affected by the variations in reference gene expression levels.

An ideal reference gene should exhibit negligible variation despite the presence of the differing experimental interventions (Vandesompele et al., 2002a). Nevertheless, studies have shown that biological differences and experimental conditions can alter reference genes' stability (Otto et al., 2020, Uppalapati et al., 2025, Zamani et al., 2020, Ho and Patrizi, 2021a). Factors such as tissue composition, dietary manipulations, genotype, and disease state can make alterations in the expression of commonly used housekeeping genes (Xu et al., 2018a, Bruckert et al., 2016, Xu et al., 2018b, Smith et al., 2024). Consequently, using unstable reference genes for the normalization of RT-qPCR data may cause inaccurate interpretations of target mRNA expression levels, which could potentially result in masking significant biological changes or producing misleading alterations. For this reason, reference gene validation under targeted experimental conditions is a very important step for identifying the most stable options for tissues and conditions under investigation and is recommended by MIQE guidelines (Bustin et al., 2009).

Additionally, this step is particularly crucial for brain studies. The brain consists of different brain regions that exhibit heterogeneity at the anatomical and functional levels. These distinct brain regions show differences in cellular organization and transcriptional diversity (Sugino et al., n.d.). Therefore, a commonly used reference gene in one brain region may not be the most suitable one for other brain regions. Moreover, experimental factors may differentially affect the transcriptional stability of reference genes across brain regions (Boda et al., 2009a).

Apoe^-/-^ mice are widely used as an animal model in atherosclerosis research. When they are given a Western diet with high cholesterol content, Apoe^-/-^ mice display increased circulating cholesterol levels accompanied by metabolic disturbances (Getz and Reardon, 2016). Since altered cholesterol metabolism is closely associated with disruption in blood-brain barrier integrity and neuroinflammation, this animal model has also been used to study the molecular consequences of lipid dysregulation in the brain (Janssen et al., 2016).

We aimed to evaluate the expression stability of six housekeeping genes that are commonly used as reference genes: *Actb*, *Gapdh*, *Rpl13a*, *Rplp0, Hprt1,* and *Ywhaz* in the cerebral cortex, hippocampus, and hypothalamus brain regions of C57BL/6 and Apoe^-/-^ mice fed either a standard rodent chow or a Western diet known to increase circulating cholesterol levels and induce hyperlipidemia in Apoe^-/-^ mice. These reference genes were selected since they are widely used for RT-qPCR normalization and represent distinct biological functions such as cytoskeletal organization, energy metabolism, structural component of ribosomal activity, purine metabolism, and intracellular signaling (Vandesompele et al., 2002b). The most commonly used algorithms, such as comprehensive ∆Ct (Silver et al., 2006a), BestKeeper (Pfaffl et al., 2004a), NormFinder (Andersen et al., 2004a), and geNorm (Vandesompele et al., 2002c), were utilized. Comprehensive ranking values were generated by RefFinder using these four computational programs. The geometric mean of the two most stable genes was used to normalize the data and assess the stability of less stable genes across brain regions, genotypes, and diets.

By determining the most stable reference genes under these experimental conditions, this study establishes a foundation for future RT-qPCR analyses of cerebral effects associated with hyperlipidemia. In addition, our findings emphasize that the selection of stable reference genes should not be generalized across different tissues and experimental conditions, but rather should be considered an integral component of data analysis.

## 2 Methods

Six to 8 week-old male C57BL/6 and Apoe^-/-^ mice (C57BL/6.129P2-Apoetm1Unc/J mice Jackson Laboratory, Bar Harbor, Maine, and created by Nobuyo Maeda, University of North Carolina) were used in the experiments. The subjects were given either a standard chow diet (4.5% fat) or a Western diet (0.21% cholesterol, 21% butterfat) (Cat#TD.88137/E15721, Ssniff-Spezialdiäten, Soest, Germany) for 16 weeks, known to induce hyperlipidemia in Apoe^-/-^ mice (Onat et al., 2019). At the end of week 16, brain tissues were collected and the cerebral cortex, hippocampus, and hypothalamus were isolated, snap-frozen in liquid nitrogen, and stored at −80°C until further analysis. All subjects were provided food and water *ad libitum* and housed in environmentally controlled conditions with a 12 h light/dark cycle, 40%-60% humidity levels, and room temperature (20–25°C). The health status of the subjects was scanned regularly by the researchers and a veterinarian. Bilkent University Local Animal Ethics Committee (HADYEK) approved all animal experiments conducted in this study with the approval date: Nov 26, 2020, and no: 2020/18.

### 2.1 RNA Isolation

Snap-frozen brain samples were homogenized in Phosphate Buffered Saline (PBS) solution. Trizol reagent (15596018, Invitrogen, Carlsbad, CA, USA) was used to isolate total RNA according to the manufacturer’s instructions (Sasik et al., 2020). Next, the TURBO DNA-free kit (AM1907, Invitrogen) was used to eliminate any possible DNA contamination. The concentrations and purity of RNA samples were measured using a NanoDropTM 2000/2000c Spectrophotometer (ThermoScientific).

### 2.2 RT-qPCR

iScript cDNA synthesis kit was used to synthesize cDNA (1708891, BioRad, Hercules, CA, USA) and 500 ng DNase-treated RNA was used for reverse transcription. cDNA samples were diluted 4-fold, and 2 µl of the diluted cDNA was used for RT-qPCR experiments. LightCycler 480 SYBR Green I Master mix solution (04887352001, Roche, Mannheim, Germany) was utilized according to the manufacturer‘s instructions. All RT-qPCR experiments were performed in the Roche LightCycler 480 System (Roche, Basel, Switzerland). The final primer concentration was 0.5 µM for each forward and reverse primer. PCR cycling conditions were as follows: pre-incubation at 95°C for 10 min, followed by 45 cycles of amplification consisting of 95°C for 10 s**,** 60°C for 15 s, and 72°C for 10 s**.** Six commonly used reference genes for the RT-qPCR normalization of the brain tissues were studied: *Actb, Gapdh, Hprt1, Rpl13a, Rplp0,* and *Ywhaz* (Xu et al., 2018c, Fan et al., 2020, Ho and Patrizi, 2021b) (Table 1). The primer sets used in this study were either selected from previous publications or newly designed using NCBI Primer-BLAST (RRID: SCR_003095) for mouse-specific target sequences. Primer efficiency was evaluated by constructing standard curves from serial cDNA dilutions (Figure S1). Primer specificity was assessed by melt-curve analysis following the amplification (Figure S2). The size of the amplicons was confirmed by agarose gel electrophoresis of PCR products. No-template controls (NTC) were included for each primer set to rule out the presence of contamination. Additionally, the distribution of Cq values was shown for each primer pair to verify that reference genes fell within the appropriate quantification interval. Each sample was run in duplicate technical replicates. Three biological replicates were used for the hypothalamus and cerebral cortex brain regions, and five biological replicates were analyzed for the hippocampal brain region.

**Table 1:** Primer sequences and amplification characteristics of the reference genes used in this study.

**Gene**	**Forward Primer 5′–3′**	**Reverse Primer 5′–3′**	**Amplicon length (bp)**	**GC content (F/R) (%)**	**Efficiency (%)**	**R**^**2**^
*Actb*	TTCGTTGCCGGTCCACACCC	GCTTTGCACATGCCGGAGCC	90	65/65	106.2	0.99
*Gapdh*	GTGAAGGTCGGTGTGAACG	GGTCGTTGATGGCAACAATCTC	97	58/50	116.2	0.99
*Hprt1*	CAGTCCCAGCGTCGTGATTA	TGGCCTCCCATCTCCTTCAT	168	55/55	113.3	0.99
*Rpl13a*	AGCCTACCAGAAAGTTTGCTTAC	GCTTCTTCTTCCGATAGTGCATC	129	43/48	102.4	0.99
*Rplp0*	GTCCTCGTTGGAGTGACATCG	GTCTGCTCCCACAATGAAGC	94	60/43	109.1	0.99
*Ywhaz*	GGTCTGGCCCTCAACTTCTC	TGGCTTCATCGAAAGCTGTTT	94	60/43	102.9	0.99

### 2.3 Gene stability analysis

RefFinder online tool (https://blooge.cn/RefFinder/) was used to assess the expression stability of selected reference genes. RefFinder integrates four commonly used algorithms (Comparative deltaCt (Silver et al., 2006a), BestKeeper (Pfaffl et al., 2004a), NormFinder (Andersen et al., 2004a), and geNorm (Vandesompele et al., 2002d)) to generate gene stability rankings. The raw quantification cycle (Cq) values were imported to RefFinder, which produces different stability rankings for each gene. A lower ranking value indicates higher expression stability. A comprehensive ranking was then calculated by taking the geometric mean of the generated stability rankings. The geometric mean of the two most stable genes identified for each brain region was used to normalize RT-qPCR data. This normalization allowed for the evaluation of whether the less stable genes were affected by genotype and diet.

### 2.4 Gene expression analysis and statistics

The most stable two genes for each brain region were selected according to comprehensive rankings (Vandesompele et al., 2002c). Delta threshold cycles (∆Ct) were calculated as Cq (the least stable genes) – Cq (geometric mean of the two most stable genes). ∆∆Ct values were measured as the ∆Ct of the target gene – average ∆Ct of all samples. The fold change expression was calculated as 2^(-∆∆Ct), and fold change values were log2 transformed for statistical analysis (Sasik et al., 2020).

Statistical analyses were performed using IBM SPSS Statistics version 31.0.0.0 (117) (IBM Corp., Armonk, NY, USA). The assumptions of a normal distribution and homogeneity of variance were checked with the Shapiro-Wilk and Levene tests. When the assumptions were met, a two-way ANOVA with factors of diet and genotype was performed. Statistical significance was set as *p < 0.05.

## 3 Results

**Fig. 1:**
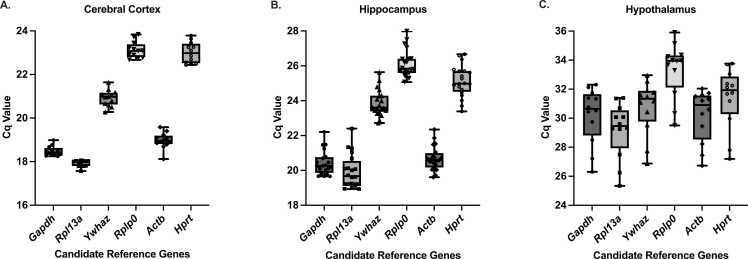
Distribution of Cq values of candidate reference genes across brain regions. Box-and-whisker plots show Cq value distributions of Gapdh, Rpl13a, Ywhaz, Rplp0, Actb, and Hprt in the cerebral cortex (A), hippocampus (B), and hypothalamus (C). Boxes indicate the interquartile range, the horizontal line within each box represents the median, and whiskers indicate the minimum and maximum values.

The distribution of Cq values was determined for the reference genes across samples in the cerebral cortex, hippocampus, and the hypothalamus (Fig. 1), and descriptive statistics are given in Table S1. All reference genes showed expression levels within the appropriate Cq range for all brain regions. However, Cq values and their variation were different across the reference genes and brain regions.

The average Cq values were ranged from 17.9 to 23.1 with relatively lower standard deviation, indicating minimal variability among individuals in the cerebral cortex brain region. The mean Cq levels ranged from 19.9 to 26.1 in the hippocampal brain region reflecting slightly greater variability. The hypothalamus brain region showed even higher Cq values ranged from 29.2 to 33.3, indicating the highest variability and standard deviation compared to the cerebral cortex and hippocampus. These results suggest region-specific differences in expression levels and variability across reference genes.

**Fig. 2:**
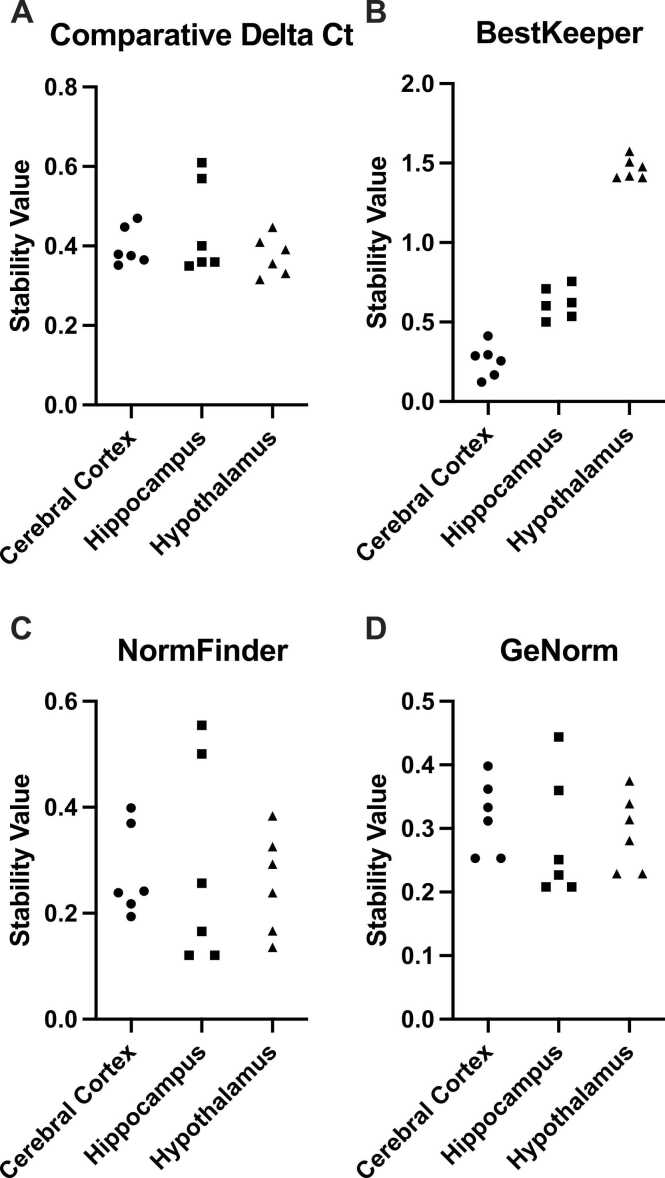
Reference gene stability for the cerebral cortex, hippocampus, and hypothalamus. C57BL/6 and Apoe^-/-^ mice were fed either chow or the Western diet for 16-weeks, which differentially increased circulating cholesterol levels and is known to induce hyperlipidemia in Apoe^-/-^ mice. (A) Comprehensive Delta Ct, (B) BestKeeper, (C) NormFinder, (D) geNorm. A lower stability value indicates a higher stability for the reference genes. The most stable brain region was the cerebral cortex, whereas the most unstable brain region was the hypothalamus.

Six different reference genes (*Actb, Gapdh, Hprt1, Rpl13a, Rplp0, Ywhaz*) were assessed to check their expression stability in three distinct brain regions; the cerebral cortex, hippocampus, and hypothalamus of the hyperlipidemic mouse model using the RefFinder online tool. Fig. 2 shows the stability values derived from the RefFinder online tool. The stability values were respectively similar for the comprehensive ∆Ct, NormFinder, and geNorm algorithms. However, the hypothalamus exceeded the cut-off value (standard deviation > 1) in the BestKeeper method. The cerebral cortex was the most stable brain region studied, whereas the hypothalamus was the least stable for these genes.

**Fig. 3:**
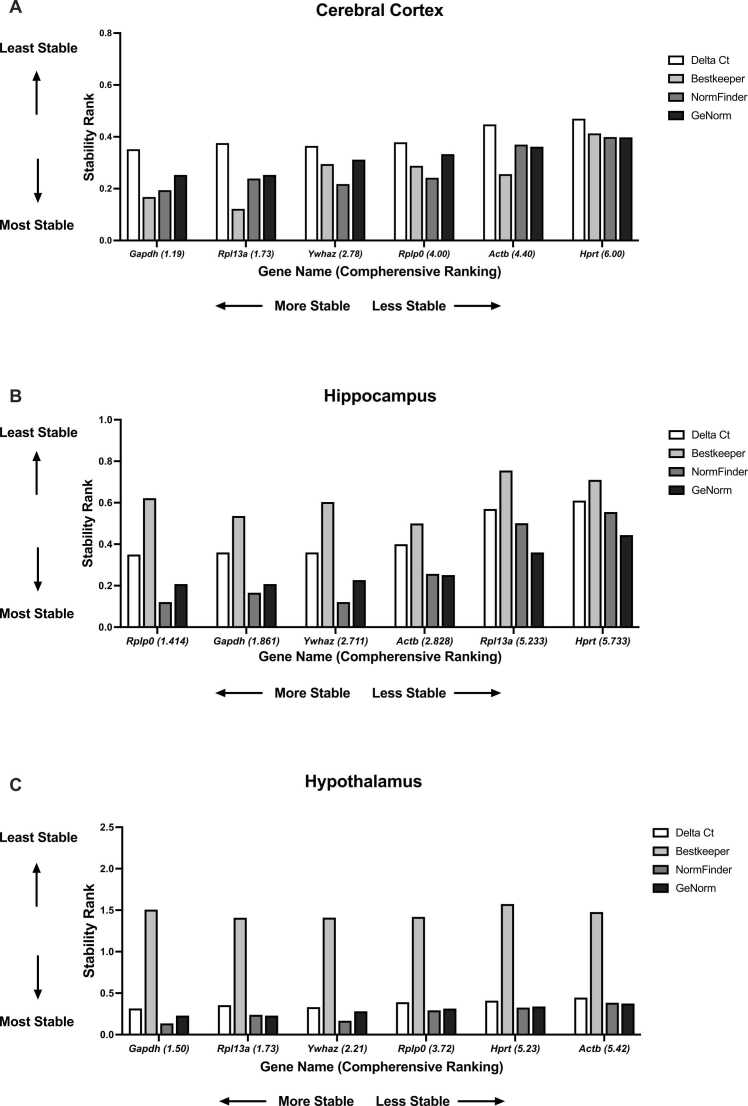
Stability ranks of the reference genes. (A) cerebral cortex, (B) hippocampus, (C) hypothalamus. The RefFinder online tool for comprehensive ∆Ct, BestKeeper, NormFinder, and geNorm methods were used for all reference genes in different brain regions. Their stability ranking was given on the y-axis. A lower stability ranking indicates higher stability for the reference genes. Comprehensive ranking values by RefFinder were provided on the x-axis next to the corresponding gene name.

The most stable genes were *Gapdh*, *Rpl13a*, and *Ywhaz* in the cerebral cortex, according to the comprehensive ranking by RefFinder (Fig. 3A). The least stable genes were *Hprt1*, *Actb*, and *Rplp0* (Fig. 3A). The most stable reference genes were determined as *Gapdh*, *Rpl13a*, and *Ywhaz* by geNorm, NormFinder, and comprehensive ∆Ct computational programs, while *Hprt1*, *Actb*, and *Rplp0* were determined as the least stable genes by the same algorithms. BestKeeper suggested *Rpl13a, Gapdh, and Actb* as the most stable genes, whereas *Hprt1*, *Ywhaz*, and *Rplp0* were the least stable ones (Fig. 3A).

The stability ranking was different for the hippocampus. *Rplp0*, *Gapdh*, and *Ywhaz* were the most stable reference genes according to the comprehensive ranking by RefFinder, and *Hprt1*, *Rpl13a*, and *Actb* were the least stable ones (Fig. 3B). Regardless of the differing order for each algorithm, geNorm, NormFinder, and comprehensive ∆Ct computational programs identified *Gapdh*, *Rplp0*, and *Ywhaz* as the most stable. Additionally, *Hprt1*, *Rpl13a*, and *Actb* were determined as the least stable genes by the same algorithms. BestKeeper identified *Actb*, *Gapdh*, and *Ywhaz* as the most stable genes and *Rplp0*, *Hprt1*, and *Rpl13a* as the least stable (Fig. 3B).

Similar to the cerebral cortex, *Gapdh*, *Rpl13a*, and *Ywhaz* were identified as the most stable housekeeping genes based on the comprehensive ranking from RefFinder, geNorm, NormFinder, and comprehensive ∆Ct computational programs, even if the order was different for each algorithm. Moreover, *Actb*, *Hprt1,* and *Rplp0* were identified the least stable genes, although the order was different (Fig. 3C).

The next step was to analyze the impact of diet and genotype on the unstable genes. For each brain region, the geometric average of the two most stable reference genes was calculated and used to normalize the RT-qPCR data for unstable ones.

**Fig. 4:**
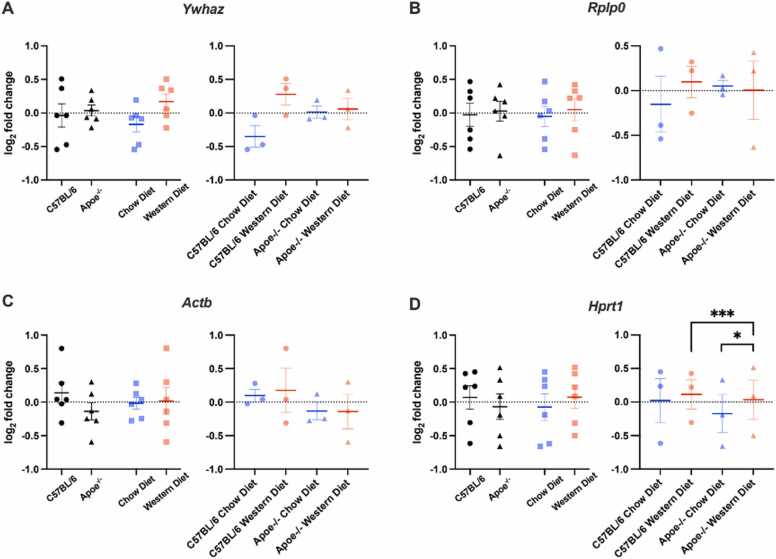
Relative gene expression levels in the cerebral cortex. (A) *Ywhaz*, (B) *Rplp0*, (C) *Actb*, (D) *Hprt1.* The effect of genotype and diet was assessed. The geometric mean of *Gapdh* and *Rpl13a* reference genes was used to normalize the less stable genes of the cerebral cortex. *: *p* < 0.05, ***: *p* < 0.001, Error bars: + /-SEM**.**

A two-way ANOVA was used to analyze whether less stable reference genes were affected by diet or genotype. Our analysis revealed no significance for *Ywhaz* (genotype: F(1,8)= 0.454, *p* = 0.519; diet: F(1,8)= 0.274, *p* = 0.615; interaction: F(1,8)= 0.124, *p* = 0.734) (Fig. 4A), *Rplp0* (genotype: F(1,8)= 0.011, *p* = 0.919; diet: F(1,8)= 0.045, *p* = 0.837; interaction: F(1,8)= 0.794, *p* = 0.399) (Fig. 4B), *Actb* (genotype: F(1,8)= 0.388, *p* = 0.551; diet: F(1,8)= 0.116, *p* = 0.742; interaction: F(1,8)= 0.770, *p* = 0.406) (Fig. 4C) were detected. *Hprt1* expression levels were not affected by diet (F(1,8)= 0.297, *p* = 0.600 (Fig. 4D) and genotype (F(1,8)= 2.519, *p* = 0.151) (Fig. 4D); however, the interaction was statistically significant (F(1,8)= 13.173, *p* = 0.007) (Fig. 4D). *Hprt1* levels (*p* = 0.018) were significantly increased in Apoe^-/-^ mice fed with the Western diet compared to Apoe^-/-^ mice fed with chow diet. Furthermore, C57BL/6 mice had significantly higher *Hprt1* levels (*p* = 0.006) when they were fed with a chow diet compared to the Western diet fed Apoe^-/-^ mice.

**Fig. 5:**
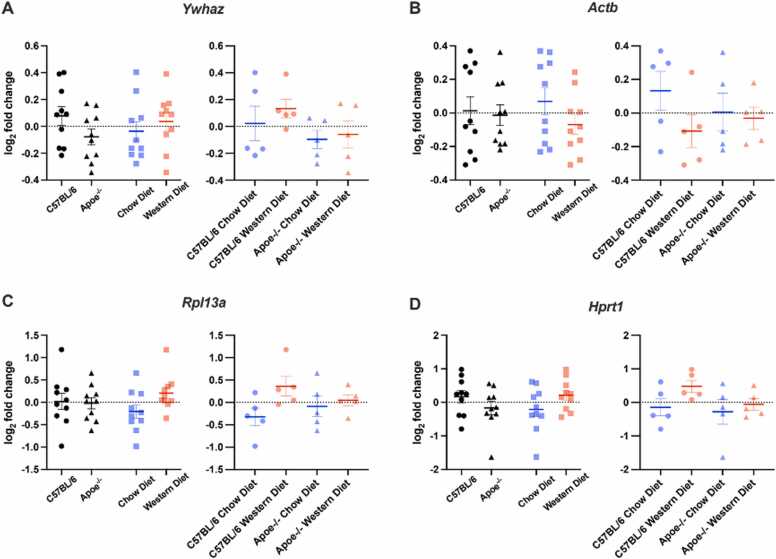
Relative gene expression levels in the hippocampus. (A) Ywhaz, (B) Actb (C) Rpl13a (D) Hprt1. Data showed the impact of genotype and diet on the reference genes in the hippocampus. The geometric mean of Gapdh and Rplp0 reference genes was used to normalize the less stable genes of the hippocampus. *: p < 0.05, Error bars: + /-SEM.

Our analysis showed no significance for *Ywhaz* (genotype: F(1,16)= 2.763, *p* = 0.116; diet: F(1,16)= 0.526, *p* = 0.479; interaction: F(1,16)= 0.084, *p* = 0.776) (Fig. 5A), *Actb* (genotype: F(1,16)= 0.065, *p* = 0.802; diet: F(1,16)= 1.898, *p* = 0.187; interaction: F(1,16)= 1.036, *p* = 0.324) (Fig. 5B), *Hprt1* (genotype: F(1,16)= 1.732, *p* = 0.207; diet: F(1,16)= 2.733, *p* = 0.118; interaction: F(1,16)= 0.623, *p* = 0.44) (Fig. 5D) were detected. *Rpl13a* expression levels were not affected by genotype (F(1,16)= 0.044, *p* = 0.836) (Fig. 5C) and there was no interaction between genotype and diet (F(1,16)= 1.946, *p* = 0.182) (Fig. 5C); however, even if there was no statistically significance (F(1,8)= 4.437, *p* = 0.051) (Fig. 5C), there was a increasing tendency for *Rpl13a* expression levels in the Western diet.

**Fig. 6:**
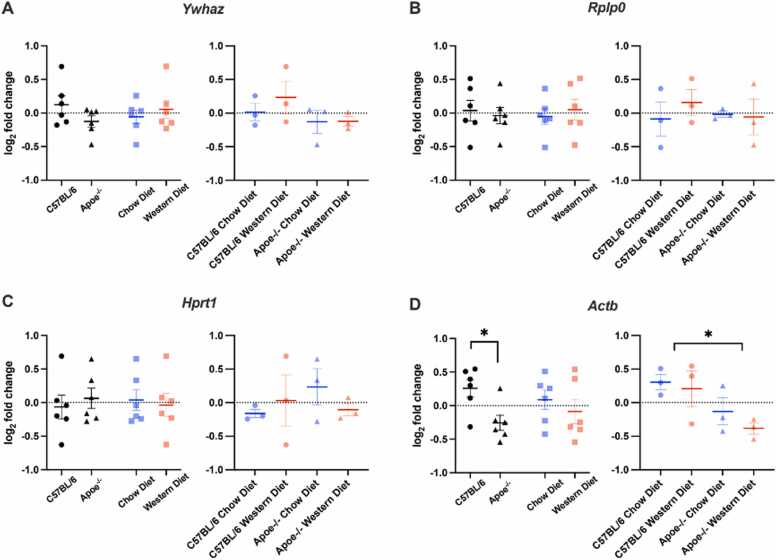
Relative gene expression levels in the hypothalamus. (A)Ywhaz, (B) Rplp0, (C) Hprt1, (D) Actb. Genotype and diet had an effect on the hypothalamus. The geometric mean of Gapdh and Rpl13a reference genes was used to normalize the less stable genes of the hypothalamus. Error bars: + /-SEM**.**

Our analysis showed no significance for *Ywhaz* (genotype: F(1,8)= 2.228, *p* = 0.169; diet: F(1,8)= 0.471, *p* = 0.512; interaction: F(1,8)= 0.398, *p* = 0.546) (Fig. 6A), *Rplp0* (genotype: F(1,8)= 0.126, *p* = 0.706; diet: F(1,8)= 0.242, *p* = 0.636; interaction: F(1,8)= 0.473, *p* = 0.511) (Fig. 6B), *Hprt1* (genotype: F(1,8)= 0.289, *p* = 0.606; diet: F(1,8)= 0.095, *p* = 0.766; interaction: F(1,8)= 1.223, *p* = 0.302) (Fig. 6C) were detected. While *Actb* expression levels were not affected by diet (F(1,8)= 0.943, *p* = 0.360) (Fig. 6D) and there was no statistically significant interaction between diet and genotype (F(1,8)= 0.179, *p* = 0.683) (Fig. 6D), there was a statistically significant main effect of genotype (F(1,8)= 8.053, *p* = 0.022) (Fig. 6D) on *Actb* levels. *Actb* expression levels were significantly decreased in the hypothalamus of Apoe^-/-^ mice.

## 4 Discussion

In this paper, we evaluated the stability of *Actb*, *Gapdh*, *Rpl13a*, *Rplp0*, *Hprt1*, and *Ywhaz,* which are different reference genes that are widely used in brain regions of the cerebral cortex, hippocampus, and hypothalamus (Uppalapati et al., 2025, Xu et al., 2018c, Schlaudraff et al., 2022). This investigation was conducted in order to demonstrate that reference genes were altered by the factors of genotype and diet. Our findings indicated that the stability rankings of these three different brain regions differed substantially. *Gapdh* and *Rpl13a* were the most stable reference genes in the cerebral cortex of the subjects; *Hprt1,* conversely, was the least stable reference gene among all algorithms. Interestingly, *Gapdh* and *Rpl13a* also had higher stability in the hypothalamus brain region, whereas *Actb* and *Hprt1* were identified as unstable reference genes. In contrast to these findings, *Rpl13a* was one of the least stable genes in the hippocampus. *Rplp0* and *Gapdh* were found to have the highest stability ranking. These differences highlight the importance of confirming the reference gene stability for different brain regions and experimental conditions.

These findings are consistent with the literature demonstrating that the stability of common housekeeping genes changes across the brain regions and experimental conditions. Previous studies have shown that the differences in reference gene expression in the mouse brain, including the hippocampus and frontal neocortex (Boda et al., 2009a). Similarly, developmental and injury-related conditions have been shown to interact with reference gene stability (Xu et al., 2018c). There are several studies that have assessed the stability of reference genes in adult mouse brain regions and under different pathological and experimental models (Xu et al., 2018d; Kang et al., 2018; Janssens et al., 2019; Pernot et al., 2010)highlight the need for region- and experimental- specific validation of housekeeping gene stability.

Overall, the present results demonstrated that the most stable brain region was the cerebral cortex, while the most unstable brain region was the hypothalamus. This distinction can result from diet and genotype factors of our experimental design. The cellular structure and physiological functions of each brain region are distinct from each other, which can differentially affect the basal expression levels of different sets of reference genes (Boda et al., 2009b). When C57BL/6 and Apoe^-/-^ subjects were fed with the Western diet, their circulating cholesterol levels were increased variably (Onat et al., 2019, Schreyer et al., 1998). The elevation of the cholesterol levels can introduce a low-grade inflammation in peripheral tissues (Onat et al., 2019). Since most of the brain regions have an intact blood-brain barrier (BBB) that protects them from the peripheral cues, these brain regions might not be affected by the changes in peripheral parameters (Abbott and Friedman, 2012). However, some brain regions, like the hypothalamus, have a leaky BBB, which makes them more prone to metabolic changes (Haddad-Tóvolli et al., 2017). Variability in reference genes for the hypothalamus can be the result of a lack of integrity of the BBB, which causes the hypothalamus to be more vulnerable to the effects of high cholesterol levels in the blood (Vagena et al., 2019). Our data showed that experimental factors such as diet or genotype might affect different tissue types to different extents, which further emphasizes the importance of the selection of stable reference genes across various brain regions.

To further assess the variability of the genes that are less stable, the two most stable reference genes were used to normalize the gene expression levels of unstable ones to further analyze the variability in reference genes. There was a significant interaction between diet and genotype on the *Hprt1* in the cerebral cortex. It was significantly increased in Apoe-/- mice fed with the Western diet compared to the chow diet group. In the hippocampus, *Rpl13a* had a tendency to increase with the Western diet. Although this change was not statistically significant, the trend may point out a diet effect on *Rpl13a* in the hippocampus. In the hypothalamus, *Actb* was significantly decreased in Apoe^-/-^ mice, which suggested a genotype effect on the *Actb* expression levels, that was limited to the hypothalamus. Overall, these results indicated that commonly used reference genes may be modulated by both diet and genetic factors, and these alterations are region-specific.

Our data also demonstrated that there are certain variances in the stability rankings for the reference genes among these four different algorithms. Although the general patterns were similar, there were some differences among the rankings constructed by Comparative deltaCt, BestKeeper, NormFinder, and geNorm. Such variations are not unexpected, since these algorithms utilize different mathematical principles to identify the stability. Comparative deltaCt uses pairwise comparisons within samples (Silver et al., 2006b), BestKeeper is based on raw Cq variation and correlation analysis (Pfaffl et al., 2004b), NormFinder estimates group variations (Andersen et al., 2004b), and GeNorm detects the most similar expression ratios across samples (Vandesompele et al., 2002e). Since these algorithms underscore different approaches to show stability, differences in ranking are predictable. For these reasons, using integrated platforms such as RefFinder (Xie et al., 2023), which integrate different outputs from several algorithms, can provide a more balanced assessment of the gene stability.

## CRediT authorship contribution statement

**Naz Mengi Camur:** Writing – review & editing, Writing – original draft, Validation, Methodology, Investigation, Formal analysis, Data curation. **Busranur Seker:** Validation, Methodology, Investigation, Formal analysis, Data curation. **Fulya Kizildag:** Validation, Methodology, Investigation, Formal analysis, Data curation. **Tulin Yanik:** Writing – review & editing, Supervision, Project administration, Investigation, Conceptualization. **Michelle M. Adams:** Writing – review & editing, Writing – original draft, Supervision, Project administration, Investigation, Funding acquisition, Data curation, Conceptualization.

## Declaration of Competing Interest

The authors declare that they have no known or perceived competing financial interests or personal relationships that could have appeared to influence the work reported in this paper.

## Footnotes

Appendix A Supplementary data associated with this article can be found in the online version at doi:10.1016/j.ibneur.2026.04.003.
