# Supplementary material for: Selection of appropriate reference genes in Apoe-/- mouse brain
Source: IBRO Neurosci Rep. 2026 Apr 24;20:653–61. doi: 10.1016/j.ibneur.2026.04.003 (PMC13141740; doi:10.1016/j.ibneur.2026.04.003)
Supplement: Supplementary file 1 — Supplementary material [file mmc1.docx]

**Supplementary Material**


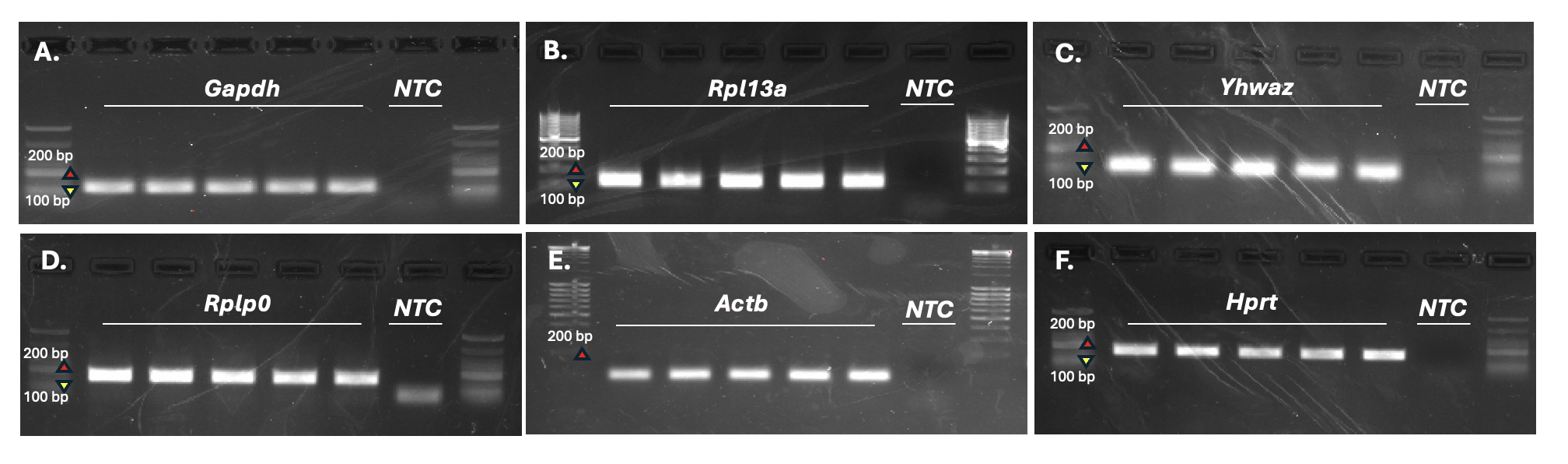


**Figure S1. Agarose gel electrophoresis of RT-qPCR amplicons of reference genes.** Representative agarose gel images show the length of the PCR products of Gapdh (A), Rpl13a (B), Yhwaz (C), Rplp0 (D), Actb (E), and Hprt (F). Each primer set showed a single band at the expected amplicon length. NTC: No-template control


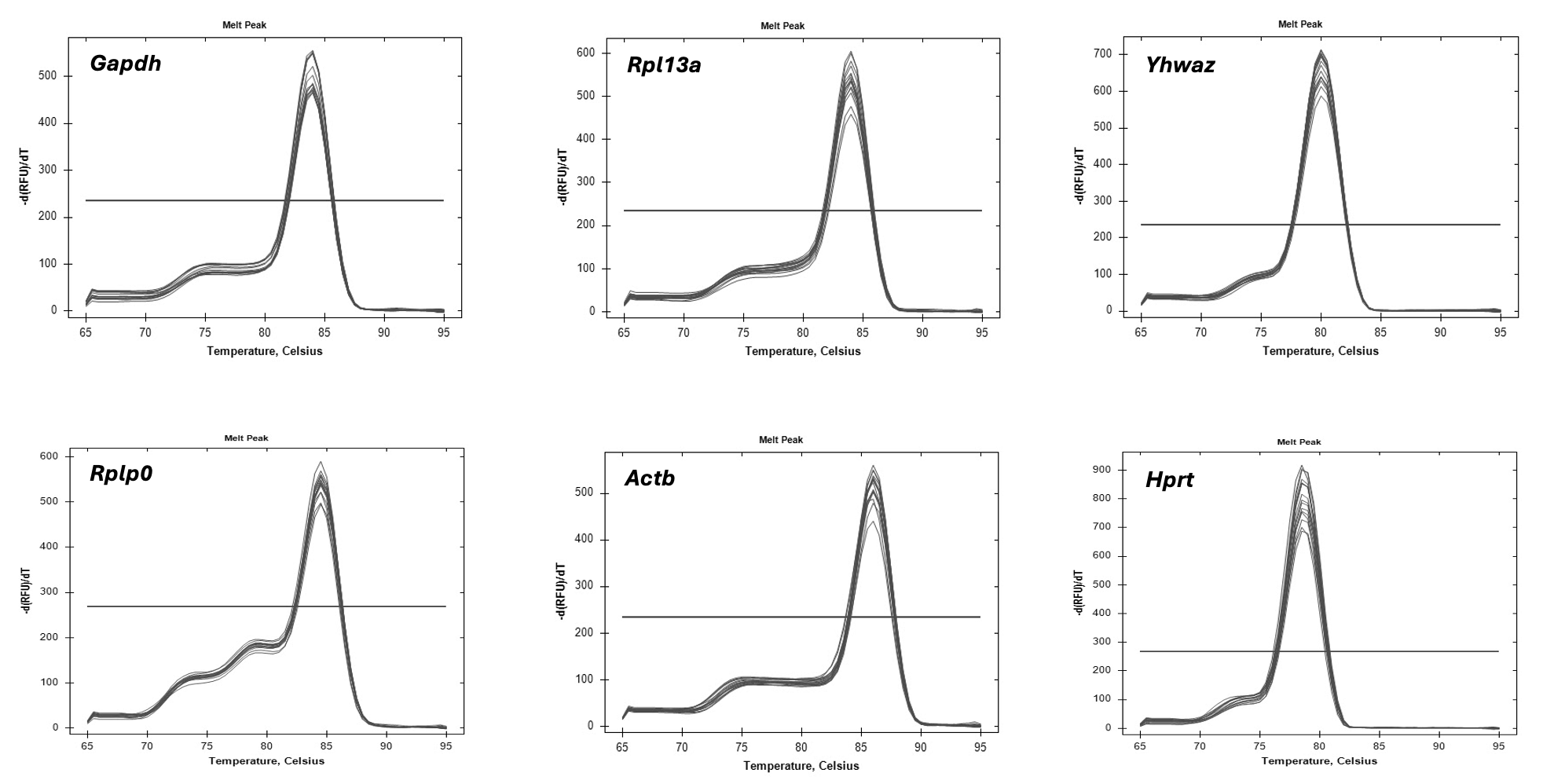


**Figure S2. Melt-curve analysis of the six candidate reference genes showed a single peak for each primer pair, indicating specific amplification.**

**Table S1. Descriptive statistics of Cq distributions for candidate reference genes in different brain regions.** Summary of the mean Cq, standard deviation (SD), minimum Cq, and maximum Cq values for the six candidate reference genes in the cerebral cortex, hippocampus, and hypothalamus.

| Gene | Brain Region | Mean Cq | SD | Min Cq | Max Cq |
| --- | --- | --- | --- | --- | --- |
| *Gapdh* | Cerebral Cortex | 18.5 | 0.22 | 18.25 | 18.99 |
| *Rpl13a* | Cerebral Cortex | 17.9 | 0.15 | 17.57 | 18.07 |
| *Ywhaz* | Cerebral Cortex | 20.9 | 0.38 | 20.28 | 21.64 |
| *Rplp0* | Cerebral Cortex | 23.1 | 0.37 | 22.67 | 23.84 |
| *Actb* | Cerebral Cortex | 18.9 | 0.37 | 18.12 | 19.59 |
| *Hprt* | Cerebral Cortex | 23.0 | 0.48 | 22.44 | 23.79 |
| *Gapdh* | Hippocampus | 20.4 | 0.69 | 19.66 | 22.21 |
| *Rpl13a* | Hippocampus | 19.9 | 0.96 | 18.94 | 22.40 |
| *Ywhaz* | Hippocampus | 23.9 | 0.76 | 22.72 | 25.64 |
| *Rplp0* | Hippocampus | 26.1 | 0.79 | 25.07 | 27.99 |
| *Actb* | Hippocampus | 20.7 | 0.69 | 19.61 | 22.35 |
| *Hprt* | Hippocampus | 24.7 | 0.89 | 23.39 | 26.67 |
| *Gapdh* | Hypothalamus | 30.2 | 1.93 | 26.31 | 32.31 |
| *Rpl13a* | Hypothalamus | 29.2 | 1.91 | 25.34 | 31.40 |
| *Ywhaz* | Hypothalamus | 30.8 | 1.85 | 26.88 | 32.96 |
| *Rplp0* | Hypothalamus | 33.3 | 1.89 | 29.50 | 35.90 |
| *Actb* | Hypothalamus | 30.2 | 1.80 | 26.74 | 32.04 |
| *Hprt* | Hypothalamus | 31.4 | 2.10 | 27.20 | 33.76 |
